# Nanotechnology-Enabled Biosensors: A Review of Fundamentals, Design Principles, Materials, and Applications

**DOI:** 10.3390/bios13010040

**Published:** 2022-12-27

**Authors:** Manickam Ramesh, Ravichandran Janani, Chinnaiyan Deepa, Lakshminarasimhan Rajeshkumar

**Affiliations:** 1 Department of Mechanical Engineering, KIT-Kalaignarkarunanidhi Institute of Technology, Coimbatore 641402, Tamil Nadu, India; 2 Department of Physics, KIT-Kalaignarkarunanidhi Institute of Technology, Coimbatore 641402, Tamil Nadu, India; 3 Department of Artificial Intelligence & Data Science, KIT-Kalaignarkarunanidhi Institute of Technology, Coimbatore 641402, Tamil Nadu, India; 4 Department of Mechanical Engineering, KPR Institute of Engineering and Technology, Coimbatore 641407, Tamil Nadu, India

**Keywords:** biosensors, nanotechnology, nanomaterials, carbon nanotubes, quantum dots, biosensing

## Abstract

Biosensors are modern engineering tools that can be widely used for various technological applications. In the recent past, biosensors have been widely used in a broad application spectrum including industrial process control, the military, environmental monitoring, health care, microbiology, and food quality control. Biosensors are also used specifically for monitoring environmental pollution, detecting toxic elements’ presence, the presence of bio-hazardous viruses or bacteria in organic matter, and biomolecule detection in clinical diagnostics. Moreover, deep medical applications such as well-being monitoring, chronic disease treatment, and in vitro medical examination studies such as the screening of infectious diseases for early detection. The scope for expanding the use of biosensors is very high owing to their inherent advantages such as ease of use, scalability, and simple manufacturing process. Biosensor technology is more prevalent as a large-scale, low cost, and enhanced technology in the modern medical field. Integration of nanotechnology with biosensors has shown the development path for the novel sensing mechanisms and biosensors as they enhance the performance and sensing ability of the currently used biosensors. Nanoscale dimensional integration promotes the formulation of biosensors with simple and rapid detection of molecules along with the detection of single biomolecules where they can also be evaluated and analyzed critically. Nanomaterials are used for the manufacturing of nano-biosensors and the nanomaterials commonly used include nanoparticles, nanowires, carbon nanotubes (CNTs), nanorods, and quantum dots (QDs). Nanomaterials possess various advantages such as color tunability, high detection sensitivity, a large surface area, high carrier capacity, high stability, and high thermal and electrical conductivity. The current review focuses on nanotechnology-enabled biosensors, their fundamentals, and architectural design. The review also expands the view on the materials used for fabricating biosensors and the probable applications of nanotechnology-enabled biosensors.

## 1. Introduction

**Figure 1:**
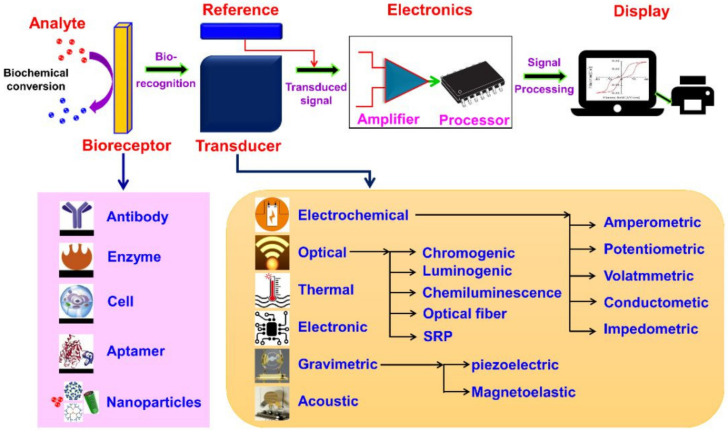
Biosensors and their components (reprinted from Ref. no. [1], copyright 2021, MDPI).

Biosensing is a traditional concept that is inherently present in many life forms, and it is used for protection against predators and harsh environments when seen through an evolutional viewpoint. Some examples of inherent biosensing in organic life forms include toxic sensibility in certain algal species, the electro-sensitive nature of sharks, and the inherent augmented offensive capability of canines [1,2,3]. As per the definition coined by the International Union of Pure and Applied Chemistry (IUPAC), a biosensor is a device that uses a precise biochemical reaction arbitrated by the immune system, isolated enzymes, organelles, or tissues for the detection of chemical compounds through the sensing of optical, thermal, or electrical signals [4]. Figure 1 shows the components of a typical biosensor.

Various combinations of biomaterials and sensor elements are possible for manufacturing a biosensor for different sensing applications. Even though a broad spectrum of materials can be used for the preparation of a biosensor, the fabrication process of a material that goes in line with the requirements of a biosensor is very complex, and this limits the manufacture of biosensors [5,6,7]. Hence, the study of compatible materials for biosensing aligned with biosensors’ requirements and the investigation of ease of fabrication is highly required. This case is more prevalent in liquid crystal (LC)-based biosensors as they are purely material-dependent biosensors. The optical properties of LC biosensors are greatly influenced by the reorientation of the aligned molecules which in turn depends on the physical and chemical surroundings of the biosensor; LCs are more commonly used for fabricating biosensors. It has been stated in various studies that LC-based biosensors find their applications in sensing novel properties [8,9,10].

Researchers have found that the LC biosensors are very interesting lately owing to the new avenues created by biosensors used for novel applications. As LC-based biosensors depend purely on molecular arrangement, they have been in the limelight for the past two decades, surpassing conventional biosensors for their utilization in a broad range of applications owing to their enhanced sensitivity [11,12]. When the light is made to go in a direction similar to the sample molecules in a polarizing optical microscope (POM) under which the LC was placed in vertical alignment, it would be blocked by the polarizer rendering a dark pattern. On contrary, if the analyte was kept along with the biosensor and observed in the POM, a parallel or random angular arrangement in oblique alignment was observed while the incident light falling over the LC material fragmented into two polarized lights in a linear direction. Such optical anisotropy of the LC materials induced rapid sensitivity and ease in visualization of the response in LC-based biosensors [13,14].

Electro-chemical biosensors are another class of biosensors that are typically used on biological analytes. These biosensors react with the recognition elements of the biological analytes and an electrical signal is produced for the transduced chemical response. These types of biosensors find their major application in environmental, clinical, quarantine, and pharmaceutical fields owing to various advantages such as miniature and portable technology compatibility, high sensitivity, low cost, and faster response [15,16]. Many of the commonly used commercial electro-chemical biosensors such as DNA hybridized biosensors, immuno-sensors and enzyme-based biosensors use biological sensing materials which possess high selectivity and sensitivity rates. Despite various advantages, electro-chemical biosensors have been built with some limitations including the high cost of the biomolecules, being highly susceptible to different environmental conditions such as oxygen content, devitalizers, pH, and temperature, lower long-term stability, complex immobilization procedure, and instability towards certain chemicals. In order to dodge the above disadvantages, electro-chemical biosensors have been built using elements with nonbiological recognition, such as transition metal oxides (MXenes) which have been used majorly during recent times owing to their outstanding stability, high selectivity, and sensitivity [17,18,19]. The current review focuses on nanotechnology-based biosensors, the materials used for the fabrication of nanobiosensors, and their potential applications. This article elaborates on the challenges faced during the full-scale implementation of biosensors and their prospects.

## 2. Nanotechnology—An Overview

Nanotechnological advances have paved the way for developing devices at the nanoscale level using various nanomaterials which directly interact and are in contact with the biomolecules or analytes for which the biosensors are intended to be used. Such biosensors have many stand-alone properties including customized magnetic, electrical, and optical properties, enhanced electrical conductivity, high sensitivity, and a low response time when compared with the traditionally used biosensors. Hence, such biosensors can be used in different bioengineering applications such as drug delivery applications [15,16]. Nanotechnology has been proven to be influential in medical fields such as disease detection with the aid of resonance, electrochemical, magnetic, electromechanical, thermal, and optical methods [17]. It can also be stated that integration of nanotechnology in the field of biosensing can offer various merits including a large surface-to-volume ratio, manifestation of biological transduction and signaling mechanisms, and electro-chemical properties [18].

In recent times biosensors have witnessed huge advancements owing to the extensive advents in transducers, nanotechnology, and signal amplifying techniques. Nevertheless, biosensors are characterized by inherent irregular signal noises. A few biosensors are highly dependent on aptamers or antibodies as bioreceptor molecules and due to this, their shelf-life is affected resulting in poor sensing stability. Commercialization of biosensors is also restricted by the reliability and accuracy of many of the modern biosensors [19,20]. Researchers are trying deeply to overcome this difficulty in many possible ways to enhance the performance of biosensors. Various studies have focused on the machine learning (ML) approach for analyzing sensing data. ML paves the way for the biosensors to abduct with their currently prevailing challenges by turning the typical biosensor into an intelligent biosensor and this works in such a way that it predicts the analyte concentration or species based on a decision algorithm. In a few other studies, chemometrics was given as a prime focus to analyze the response of a biosensor. Chemometrics is an extensively used chemical analyzing technique that uses mathematical or statistical methods to analyze and render maximum chemical data from the analysis of chemical information and to optimize and select the experimental design to bring out the optimal process of measurement [2,21,22].

It has been stated in many studies that ML can effectively process big sensing data in the form of complex samples or matrices. Some advantages of the ML method of chemical analysis include the rendering of data from low-resolution and noisy sensing data that may also have overlapped with one another. Besides these advantages, when ML methods are deployed on a full scale, the hidden relations existing within the analyte parameters and signals can be discovered with the aid of exploring the interrelations between the bio-events and the signals and through various data visualization methods. ML methods are usually used to interpret the raw form of sensing data obtained from the biosensor in the following ways:

- Categorization—Signals obtained from the sensing data are classified into different types using various algorithms depending on the type of target analyte.
- Anomaly detection—The operating conditions of a biosensor and the sample matrix significantly affect the functions of a biosensor and the contaminations interfere with the sensing signal if the biosensor is deployed in the target site. In such cases, the ML algorithm checks the correctness of the obtained signal, and if found incorrect, the signals which are interfered by the biofouling are corrected to improve the sensor performance.
- Reduction of noise—Sensed signals are commonly embedded with noise. If the sensed signal is interfered with by electrical noise, the signal changes in a few seconds or minutes which makes it shift to a sub-second timeline. If ML models are trained to detect noise, the sensing signal accuracy can be substantially improved.
- Pattern recognition and object identification—Then signals obtained by the biosensors can be effectively and easily interpreted through the discovery of latent patterns and objects with the aid of ML algorithms [23,24].

## 3. Fundamentals

### 3.1. Biosensors

To monitor biological reactions, biosensors have been used to analyze the interaction and transform it into an electrical impulse. Electro-chemical, fiber optic, piezo, sound, and thermal transducers along with biological components including enzymes, DNA, RNA, metabolites, cells, and oligos are combined in biosensors [25,26]. Basically, a biosensor is an analytical tool that detects and responds to changes in biological systems by producing an electrical impulse. Enzymes, tissues, bacteria, cells, acids, and so on are all examples of biological processes. The voltage or current would be the transducer’s signal [27,28,29], based on the kind of enzymes and the raw material utilized in the synthesis of organic compounds.

The major strength of biosensors is their capacity to transform biological interactions into a type of electric signal that can be detected and quantified. It is also crucial to focus on the effective analysis of minute variations throughout the biological processes when different biomolecules combined [30,31]. Based on the numerous advantages, biosensors are now being invented for their usage in the accurate diagnosis of infections and the evaluation of food standards, as well as other applications in the environmental sector. In medical field, biosensors have been developed to detect tumors, viruses, pollutants, and biomarkers so as to diagnose diseases at an early stage [32,33]. However, biosensors have become more significant because of their advantageous properties, such as cheap production costs, quick reaction times, mobility, and the capability to measure biological materials at a tiny scale with a high level of proficiency and sensitivities [34,35].

It has taken a long time for biosensor technology to advance for the study of other physical components. To summarize, biosensors have numerous sections or components, including the analyte, bioreceptor, transducer, electronics, and reader display. In most biosensors, the reader display is connected to the electronics or signal processors which are required to interpret the data [36,37]. As each biosensor operates on different working principles, every reader must be conceived and built from scratch. Often, this is the costliest aspect of developing sensors. As the sensor evaluates the consumer in response to different stimuli, the transducer records the information and converts the stimulus into an electrical impulse that can be measured as output data [38].

Biosensors find their usage in a wide variety of applications, including waste management, health monitoring, agricultural experimentation, forensics, biological testing, and water quality control. Biosensory medical clothing is often used for overall health observation, diseases diagnosis, and clinical evaluation [39,40]. Overwhelmingly, glucose biosensors have been used to monitor and control diabetes. Blood sugar levels, a key indicator of diabetes risk, are also monitored using biosensors. Biosensors’ continued significance is shown by the fact that they enable patients to maintain their desired blood sugar levels and enable researchers to trace the disease’s ecological impact. Biosensors may speed up disease diagnosis and patient monitoring [41,42], adding value to traditional medical treatment.

### 3.2. Nanobiosensors

Nanoparticles are integrated during fabrication and the resulting biosensors are called nano-biosensors. Nanomaterials are always the most investigated and examined of these because of the wide range of bioanalytical activities they provide in fields such as bio-imaging, diagnostics, medication administration, and the treatment that they enable [43,44]. Amperometric equipment has been used to assess enzyme-based reactions, whereas fluorescent QD devices are being used for measuring the binding efficacy and immunolabeling applications that utilize conjugated nanoparticles to analyze biomolecular interactions. The inherent optical features of nanoparticles as well as the potential ability to be coupled to fluorescent markers make them a promising biosensor. Electro/chemiluminescent tests, fluorescent-based tests, and biological field-effect transistors (bio-FET) tests can end up in making CNTs, and other relevant carbon-based nanostructures including reduced graphene oxide (rGO), graphene, and graphene sheets. Fluorescent-based assays are usually detected through the quenching characteristics of graphene [45,46], and carbon nanomaterials can adopt a variety of structures depending on the method of measurement being used.

**Figure 2:**
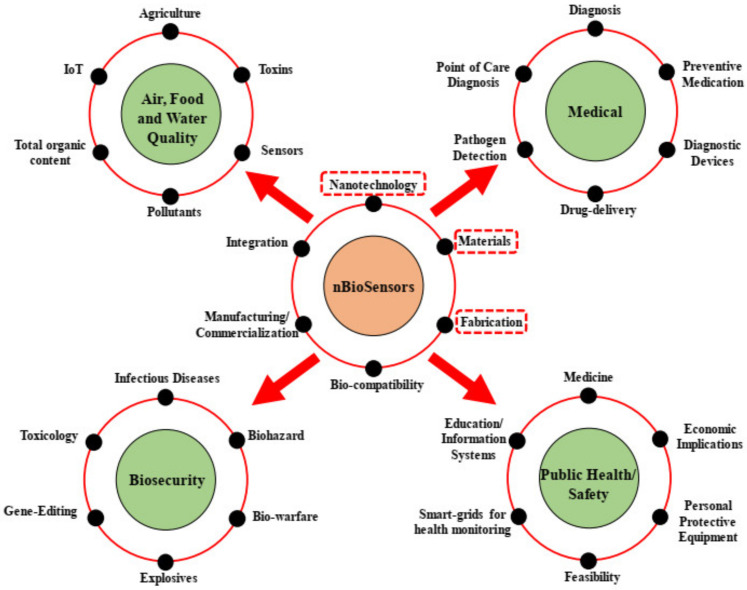
Advancements and applications of nano-biosensors (reprinted from Ref. no. [2], copyright 2021, MDPI).

Based on their size and form, which defines their fluorescence qualities, QDs provide a wide range of applications in the areas of sensors and imaging. Functionalizing QDs with polymers such as PEG and polysaccharides is required for specific identification. QDs have several advantages over conventional dyes, including a higher yield of molar extinction and quantum coefficient, a wider range of absorption and smaller spectra of emission, photo-bleach resistance, and so on [47]. They are widely utilized in FET as well as in sandwich assays. In addition to the nanostructures already described, there are several distinct nano-based biosensors that may be used for a variety of purposes. Unusual applications of nanotechnology for biosensing seems to be the development of glucose sensors for people with diabetes, the identification of antigens of HIV/AIDS, the monitoring of the bioburden of microbes in cases of infection in the urinary tract, and diagnostic tests for cancer. Therefore, nano-biosensors are indeed a useful tool in tissue engineering for either diagnosis or therapy [48,49]. Figure 2 shows the different applications of nanobiosensors.

Nanosensors, nanoprobes, and other nanoscale technologies have greatly improved the quality of chemical and biosensing structures. These nanodevices have been developed with fast reaction times and minimal power consumption in consideration. Electrochemical indications of biocatalytic processes at the conductive junction have been enhanced with the help of nanomaterials such as magnetic nanomaterials, metal and oxide nanoparticles, QDs, metallophthalocyanines, and carbon materials. Functionalized nanoparticles attached to organic molecules have been produced for their potential application in biosensors. There is a wide range of methods for synthesizing nanostructured materials and nanodevices, while the choice of the best options solely depends on the materials’ properties, the nanomaterial kind being synthesized (0D, 1D, 2D), the size of the required amount, and many more factors [50,51,52]. Especially in biosensing applications, nanostructured materials are being developed using a wide variety of physical processes [53], including physical vapor deposition (PVD), high-energy ball mixing, melt mixing, electric arc, laser ablation, and sputtering.

Nanostructures are also synthesized chemically, most notably by the sol–gel technique and inverse micelles formation. Materials with nanostructures may be synthesized by using a bottom-up and top-down technique. The bottom-up approach is the downsizing of material building blocks associated with self-assembly results to produce nanostructures. QDs and nanoparticles are created from colloidal dispersions using these techniques. The lower failure rates and more consistent elemental composition of such methods make them a suitable alternative [3,54]. Top-down methods, on the other hand, begin with the desired nanostructure and then carefully design the processing of macroscopic structures. Some examples are ball milling, extreme plastic deformation, and inductively coupled plasma (ICP) etching. The existence of very high numbers of flaws in the surface structure is, however, a fundamental downside of these techniques [55].

## 4. Architectural Design

In order to design and create a biosensor with good sensing abilities, its architectural design based on various materials must be considerably taken care of. The use of conductive polymers (CPs) and transition metal oxides may result in designing a good biosensor with enhanced capabilities. CPs have recently been synthesized by various synthesis methods such as biochemical, chemical, and electro-chemical formation techniques [56]. Various types of research has been completed in bringing out better methods for the synthesis of CPs which in turn can be used for the design of durable and reliable biosensors. It has been stated in many studies that selecting the most suitable monomer element for fabricating a CP layer with sensing capabilities significantly influences the architectural design of a developed biosensor. CPs have been characterized by the presence of delocalized π-electrons at the polymer chain backbones which makes the CPs hold some unique properties such as low ionization potential, enhanced electrical conductivity, and some other exciting characteristics [57,58,59]. Such standalone properties have allowed CPs in various significant applications such as transistors, rechargeable batteries, light-emitting diodes, electrochromic displays, biosensors, smart windows, and photovoltaic devices. CPs have been utilized lately to construct various biosensors and catalytic sensors for bioanalytics. Redox enzyme–glucose oxidase (GOx) is a glucose biosensor in which the design of the biosensor usually includes the CPs for biological element recognitions. As stated by various research works on GOx sensors, it can be used as a biocatalyst during the manufacturing of various CPs including polytiophene, polyaniline, and polypyrrole [60,61].

Certain disadvantages are associated with traditional bioanalytical techniques, such as the enzyme-linked immune sorbent assay (ELISA), including expensive biomaterials having to be used, complex analysis processes, and the techniques not being long-lasting, even though they possess numerous advantages such as accuracy in target biomolecule detection. In order to overcome this complexity, the use of affinity sensors has been recommended for various applications. Natural or artificial receptor-based biosensors, antibody-based biosensors, molecularly imprinted polymer (MIP)-based biosensors, and DNA-aptamer-based biosensors are some of the classifications of affinity sensors. Amongst these, MIP-based biosensors are considered to be effective owing to their non-dependency over expensive biological molecules and they have been built solely based on the polymeric matrix [62,63,64]. Transition metal oxides and MXenes have recently been developed (during the last decade). Two-dimensional materials for the architectural design and construction of biosensors have been developed owing to their excellent semiconducting properties, metallic conductivity, and/or a combination of both. Such unique property combinations can be used for the design and manufacture of biosensors, wearable electronic devices, and biofuel cells [65,66]. When compared with the physical properties and structural relationships of typical 2D materials such as graphene, MXenes have been found to possess similar properties and most of these materials depend on 2D transition metal carbides, nitrides, and carbonitrides. Owing to the flexibility offered by MXenes towards the design of biosensors when compared with other materials, they have been proven to be one of the ideal candidates for the design of biosensors [67,68].

## 5. Nanomaterials for Biosensors

The evolution of research on biosensors has gained attention among researchers with its potential utilization in industrial applications and scientific fields including bioinformatics, biotechnology, electronics, materials science, healthcare, medical science, and so on [69]. A biosensor is a device that can sense a biological element in contact with an analyte and transduce the biological response received into an appropriate signal through a transducer. There are many challenges in designing biosensors such as sensitivity, response time, reproducibility, and poor detection limits [70]. Among the many challenges in the development of biosensors, the major concern is the limit of detection (LOD) of biological analytes [71]. The importance of this emerging technology has been intensified to the next level with the contribution of blooming nanotechnology. Nanobiosensors have recently been a hot topic of research as nanostructures with their ultra-low dimensions showcase novel properties over their bulky counterparts. Hence, nanomaterials are being used as candidates for transducer coatings in order to achieve solicited detection at the picomolar level [72].

### 5.1. Graphene-Based Biosensors

**Figure 3:**
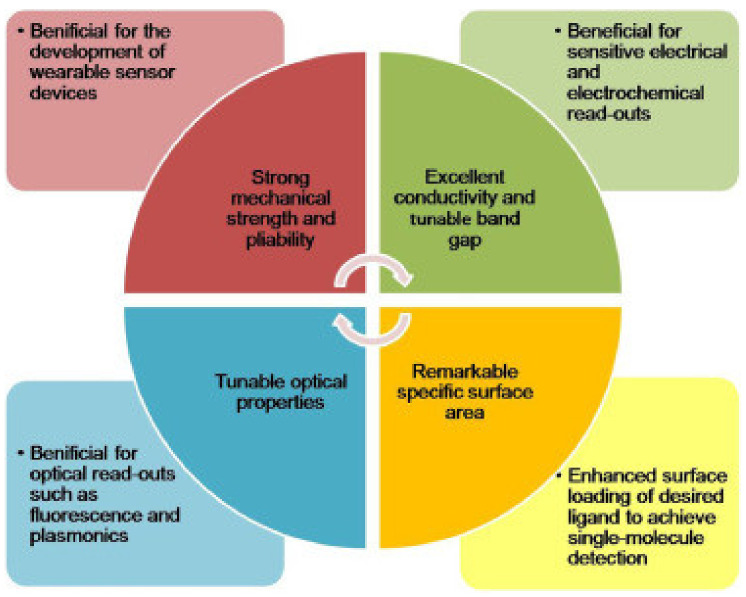
Chemical and physical characteristics of graphene and related materials (reprinted with permission from Ref. no. [73], Copyright 2021, Elsevier).

Nanotechnology praises graphene as a ‘wonder material’ owing to its extraordinary physicochemical characteristics. Characteristics such as high conductivity, low charge carrier resistance and a high surface–volume ratio make graphene an ideal candidate for designing a transducing material (Figure 3). Due to its one-atomic thickness, graphene is a 2D structural material. It is a sp^2^ hybridized form of carbon with a hexagonal lattice structure [73]. The conductivity and dimensionality of graphene sheets can make every atom easily exposed to environmental fluctuations which is very necessary for a sensing application [74]. Graphene is reported as the material with the lowest resistivity in the world, with it having a conductivity of 10^6^ s/m and resistivity of only 10^−6^ ohm.cm which is a lot lower than copper [75].

Graphene can be synthesized in a wide number of ways; however, techniques for synthesizing bulk graphene on a large scale are still lagging which obstructs its utilization in various potential applications. GO is obtained out of graphene through oxidation through several routes and GO can be further converted to rGO through the reduction of GO [76]. There are numerous physicochemical methods available to synthesize graphene and its derivatives which include rubbing graphite on a surface [77], physical exfoliation through scotch tape [78], the chemical oxidation of graphite [79], the arc discharge method, ball milling, chemical vapor deposition, and liquid phase exfoliation [80].

A few experimenters have designed an ultra-highly sensitive graphene optical biosensor (GOB) for detecting whole living cancer cells responding to the chemotherapy drug paclitaxel during drug delivery. In their work, a novel optical strategy for detecting refractive index (RI) changes has been followed with the advantage of sensing the living cells label-free. This method is also beneficial in terms of non-destructive measurements and dynamic cell monitoring free from electro-magnetic interference and surface contamination [81]. They demonstrated experimentally that the detection of ultra-small RI changes of 1.35 × 10^−7^ was made possible using a GOB with a corresponding sensitivity of 1.2 × 10^8^ mV/RIU and a response time of 260 ns. On the other hand, field effect transistor (FET)-based biosensing technology has been extraordinarily explored for instantaneous highly sensitive measurements using a tiny number of analytes [82].

Moreover, when compared to optical biosensors, FET-based biosensors eliminate the complications of enzyme labelling and do not necessitate expensive optical instruments [83]. Afsahi et al. [74] deposited high-quality single-layer graphene by the chemical vapor deposition (CVD) technique over a commercially available biosensor chip for the specific detection of an epidemic virus named Zika viral non-structural protein 1 (ZKV NS1). The deposited graphene layer over the commercial biosensor chip works based on the field effective biosensing (FEB) technology wherein the channel current and gate capacitance in the graphene transistor chip shift in accordance with the immobilization of the biological targets to the gate current. Their experiment with the cross-linkage of Zika NS1 to a graphene biosensor chip resulted in ZIKV NS1 detection at the very low concentration of 0.45 nM. A few experimenters have made some serious efforts towards fabricating a graphene FET-based immunosensor for rapid detection of the COVID-19 spike protein. In their work, researchers constructed a biosensor by immobilizing two receptor binding domains such as human angiotensin-converting enzyme 2 (ACE2) and SARS-COV spike S1 subunit protein antibody (CSAb)—OVID-19 and compared their performance. They observed that the CSAb-modified biosensor could deliver real-time detection with an LOD down to a 0.2 pM concentration with a fast response time of two minutes [83].

**Figure 4:**
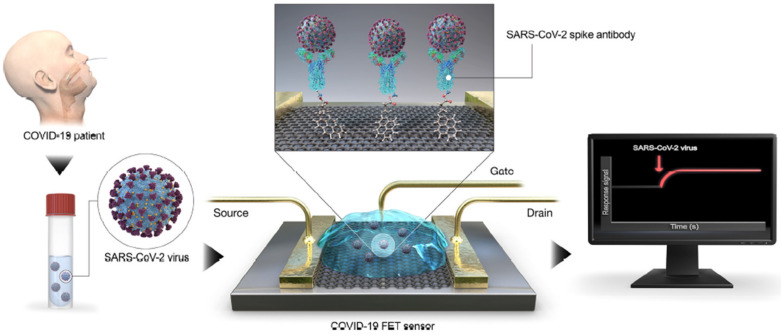
Schematic diagram of COVID-19 FET sensor operation procedure (reprinted from Ref. no. [82], copyright 2020, American Chemical Society).

Similarly, real-time detection of the COVID-19 virus from the nasopharyngeal swab specimens of affected patients using graphene-based FET biosensors has been reported by a few researchers (Figure 4). A sensor was designed which could detect the antibody through the graphene layer coating over the FET biosensor with the SARS-COV-2 spike protein with an LOD as low as 1 Fg/mL [82]. A novel concept of biological sensing through electrolyte-gated rGO FETs has been demonstrated by Aspermair and his co-researchers to identify the E7 protein of the human papillomavirus (HPV) in human saliva. HPV has been reportedly noted for causing cervical carcinogenesis through sexual transmission. They have successfully fabricated pyrine-modified rGO-based FET and achieved an LOD of 100 pg/mL (1.75 nM) for HPV-16 E7 [84].

### 5.2. Gold Nanoparticles (GNPs) in Biosensors

**Figure 5:**
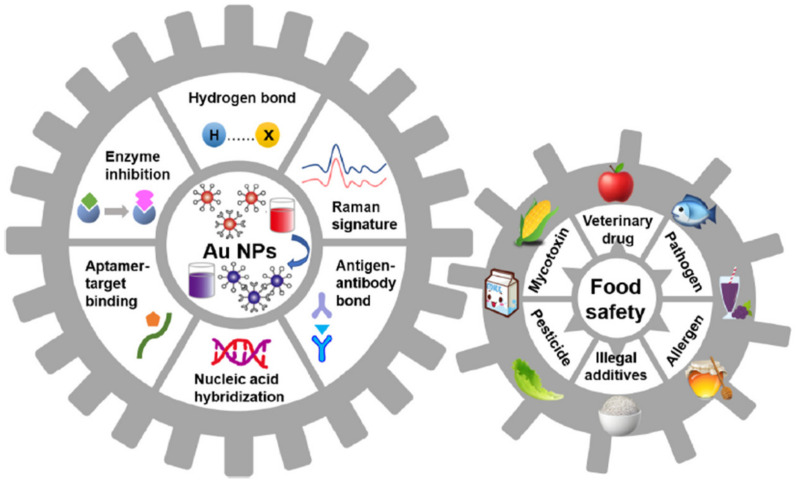
Schematic overview of different mechanisms of GNP-based biosensors for food safety detection (reprinted with permission from Ref. no. [87], copyright 2021, Elsevier).

GNPs offer an excellent platform in biosensing with their unique colorimetric property depending on their shape, size, and state of aggregation in the presence of analytes [85]. The transformation of GNPs from a monodispersed state to an aggregate state in a liquid medium induces a visually evident color change from red to blue. With a diameter between 1–100 nm, GNPs hold a high surface-to-volume ratio and high surface energy. They facilitate rapid direct electron transport between a wide range of electrodes and electro-active species. GNPs are thus utilized for biosensing applications in the fabrication of optical biosensors, electro-chemical biosensors, and piezo-electric biosensors. In optical biosensors, the changes in the light or photon output are sensed to give the appropriate signal. GNPs are utilized in optical biosensing based on their optical sensing modality and surface plasmon resonance (SPR) behavior. GNPs are known to have the ability to enhance the local electro-magnetic field enabling SPR signal amplification which can bring up a dielectric constant variation and cause luminescence from the metal–liquid surface [86]. These distinct characteristics of GNPs have been largely utilized in the biosensors’ fabrication of food safety applications in detecting various allergens and contaminants (Figure 5).

**Figure 6:**
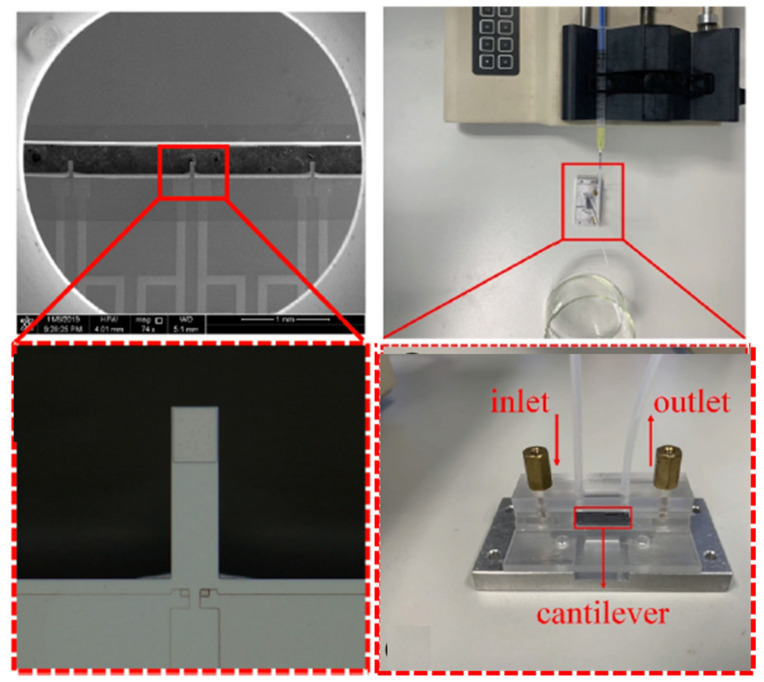
Enlarged view of the biosensor fabricated through the cantilever method and the process flow adopted for the manufacturing of the biosensor (reprinted with permission from Ref. no. [91], copyright 2021, Elsevier).

Interestingly, a few researchers have incorporated GNPs into designing micro-cantilever-based biosensors to detect protein biomarkers. They have embraced a technique of crafting a microcavity at the free end of the cantilever for a local biological reaction that facilitates in achieving high detection accuracy. The detection limit attained for the GNP-amplified biosensor was about 21 pg/mL, which is 70 times of magnitude smaller than the one without GNP amplification [88]. Yuan’s group have employed GNPs to detect DNA for allergies to peanuts, soybean, and sesame through colorimetric readouts. During the process of detection, the allergen gene releases long double-stranded DNA from the GNP surface and makes them aggregate and change to a blue color with the addition of NaCl. If the gene sample is below the threshold level, then the DNA probes would stick to the GNPs inhibiting them from aggregation and remaining in the red color. With this technique, they were able to recognize allergen gene targets with an LOD of 0.5 nM [89]. Researchers developed a GNP-based biosensor with the first-ever dual-sensing technology to rapidly detect SARS-CoV2 spikes using human saliva [90]. The developed system was able to detect SARS-CoV-2 spike antigens through electrochemical and colorimetric methods. The colorimetric assay detected SARS-CoV-2 spikes with the aid of antibody–antigen interaction, and it resulted in the accumulation of GNPs, therefore, changing the color from red to purple with an LOD of 48 ng/mL. In the electro-chemical detection method, the as-prepared probe solution is dropped on the disposable screen-printed gold electrode with a very low LOD of 1 pg/mL [90]. Figure 6 shows the enlarged views of fabricating a biosensor using the cantilever method.

A few experimenters have demonstrated the real-time detection of hepatitis B surface antigen (HBsAg) by graphene GNP hybrid biosensors. The GNPs were decorated on the graphene layer substrate through π–π interaction stacking of single-stranded DNA (ssDNA). The developed hybrid sensor had the potential to detect HBsAg down to the detection limit of 50 pg/mL [92].

### 5.3. CNTs in Biosensors

**Figure 7:**
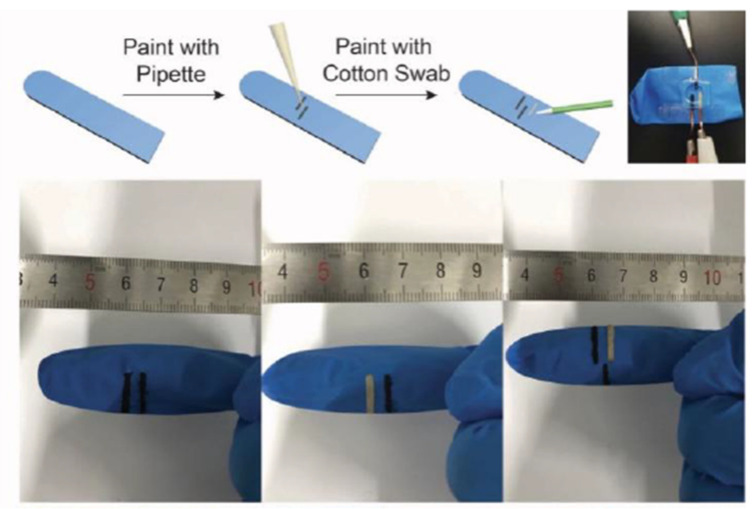
Camera images showing the process of fabrication of CNT-based biosensor-painted gloves (reprinted from Ref. no. [96], copyright 2018, MDPI).

Carbon in its allotropic form is known as CNT and is distinguishably known for its excellent mechanical, electrical, electrocatalytic, and thermal properties. It is also expressed as a rolled-up graphene layer and its properties are determined by the number of composed walls [93]. The special geometry of CNT has attracted many potential applications in the field of biosensors. Single-walled CNTs (SWCNTs) have the lowest diameter ranging between 1 nm to 2 nm, while multi-walled CNTs (MWCNTs) can have diameters up to 100 nm. Due to the large number of layers around them, they possess many inevitable defects. Double-walled CNT (DWCNT) on the other hand falls intermediately between SWCNT and MWCNT with many properties of SWCNT as well as a smaller number of defects when compared to MWCNT [94]. CNTs showcase better biosensing properties when doped with polyaniline (PANI). PANI is known for its improved redox activity which when combined with CNT could generate an amplified signal response in electrochemical biosensors. The CNT–PANI nanohybrid possesses a unique flower-like structure providing a large surface area and has been experimented for micro bacterium tuberculosis (MTB) detection [95]. The nanohybrid could deliver super sensitivity of MTB with an LOD of 0.33 fm. Moreover, the electrical properties of CNT have been employed in lactase detection from human sweat samples. A few experimenters have developed special wearable CNT-based biosensor-painted gloves for the determination of lactase present in human sweat as depicted in Figure 7. The constructed CNT/Ag/AgCl painted gloves showed sensitive detection of lactase up to a detection limit of 1.4 μm [96].

**Figure 8:**
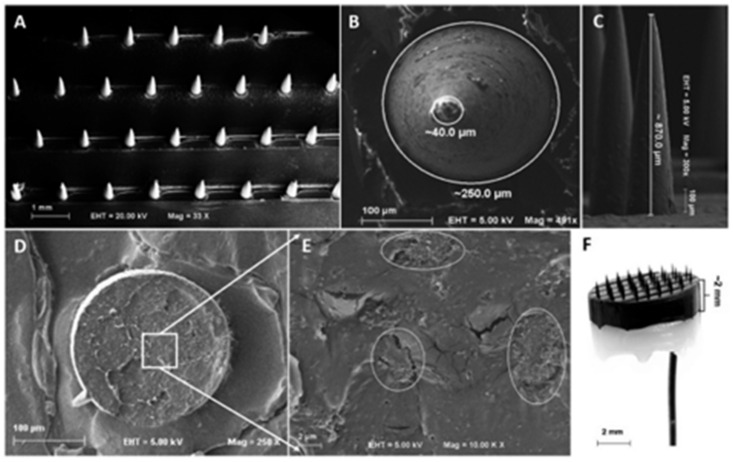
SEM images: (**A**) conical microneedles fabricated through a micromolding technique, (**B**) lateral dimensions of the microneedles, (**C**) longitudinal dimension of the microneedle, (**D**) morphology of fractured microneedle along with the stump, (**E**) enlarged fracture morphology, and (**F**) micrographic image showing the complete view of the microneedle mounted on an composite base (reprinted from Ref. no. [97], copyright 2019, American Chemical Society).

The utility of CNTs in dermal biosensing has paved the way for cost-effective health monitoring in a non-invasive manner. Some experimenters have designed a microneedle array (MNA)-based polylactic acid (PLA)/CNT composite biosensor for thermal injuries. The matrix of micro-needles with PLA provides outstanding mechanical properties for skin penetration without causing pain/bleeding and it acts as in situ in electrochemical biosensors. Electrochemical measurements in ex vivo porcine skin produced a linear current response corresponding to an LOD of 180 μm [97]. Figure 8 illustrates the process of the fabrication of biosensors and the morphology of the resulting biosensor surface.

### 5.4. QD-Based Biosensors

**Figure 9:**
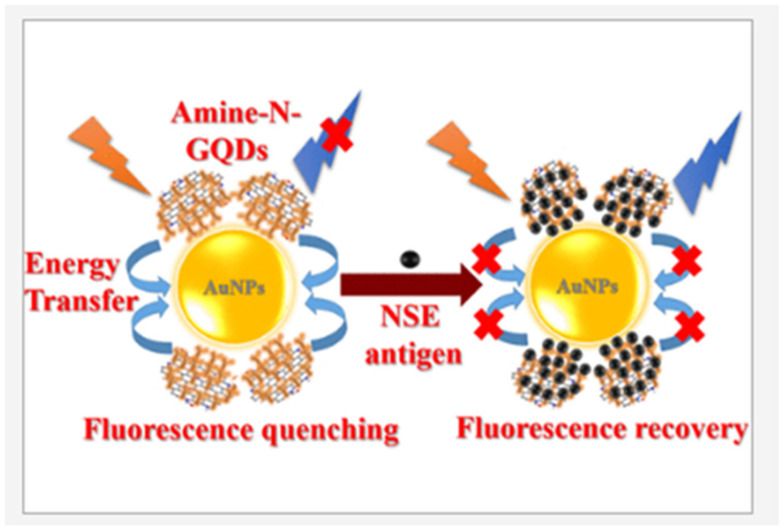
Fluorescent biosensor working mechanism for the detection of cancer biomarkers (reprinted from Ref. no. [101], copyright 2020, American Chemical Society).

QDs or the so-called fluorescent semiconductor nanocrystals are nanoparticles possessing diameters less than 10 nm. Recently, they have attracted significant interest in cutting-edge biosensing technology. The astounding optical, electronic, and size-dependent luminescent properties of QDs are much utilized by researchers in sensing applications [98]. QDs are proven to offer a high surface-to-volume ratio and extraordinary charge carrier transport properties that could support improving the performance of a biosensor. In this aspect, carbon quantum dots (CQDs) have become noteworthy in frontline sensing research works because of their striking properties such as low toxicity, higher solubility, chemical stability, and versatility [98,99]. For instance, a biosensor based on CQDs was designed by Wei’s group for the rapid detection of a carcinogenic element acrylamide (AM) from food products. CQDs were manufactured through a one-pot hydrothermal technique and coupled with ssDNA to quench their fluorescence. During the addition of AM, ssDNA formed a hydrogen bond with AM leaving behind very few free DNAs and eventually reduced the degree of fluorescence provided by CQDs. The fluorescence property of CQDs and the high affinity between ssDNA and AM were utilized in the real-time detection of AM from bread crusts resulting in an LOD of 2.41 × 10^−8^ M [99]. ZnO QD-based biosensors were developed by Kamaci’s group for the detection of cysteine in solutions. They came up with a novel strategy of developing a fluorescent probe using melamine-modified ZnO QDs. The ZnO QD-based fluorescent biosensor displayed a stronger fluorescence response toward cysteine detection with a linear range from 0.1 to 600 µm and an LOD of 0.642 µm [100]. Figure 9 shows the mechanism of fabricating a fluorescent biosensor for cancer biomarker detection.

**Figure 10:**
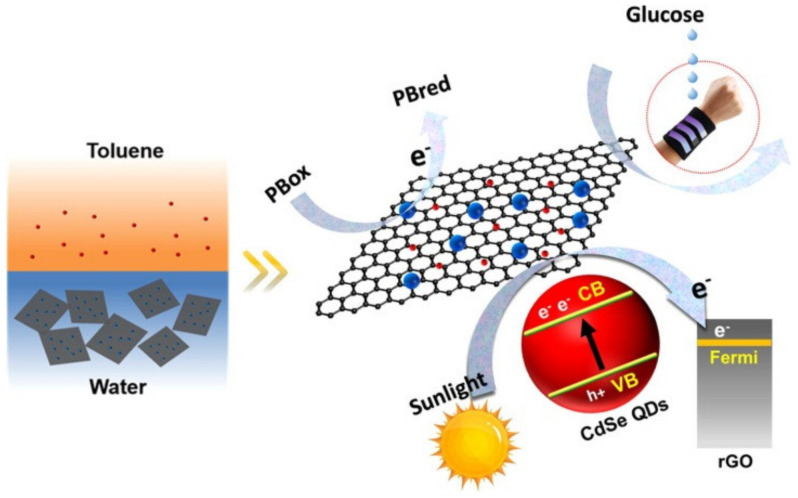
Schematic of PB–rGO–QDs film preparation through experimental method (reprinted with permission from Ref. no. [102], copyright 2022, Elsevier).

Furthermore, detecting small lung cell cancer rapidly and in a premature state has been made possible by designing biosensors with graphene quantum dots (GQDs) as energy donors while the energy receptors were GNPs [101]. The fluorescence response study carried out for lung cancer detection offered a notable LOD of 0.09 pg/mL with a response time of 17 min and a larger detection range from 0.11 pg/mL to 1002 ng/mL. A simple and low-cost textile-based wearable sensor for glucose and H_2_O_2_ sensing has been developed by some researchers as depicted in Figure 10. A fabric-based nanofilm technology was developed by integrating Prussian blue (PB) with CdSe QDs and rGO QDs through oil–water self-assembly engineering over a flexible ITO substrate. The films expressed excellent electro-chemical sensing activity with a high sensitivity of 53.8 µA mM^−1^ cm^−2^ for H_2_O_2_ and 37.24 µA mM^−1^ cm^−2^ for glucose, respectively [102].

### 5.5. Cell-Based Biosensors

Among the biosensors that are being used as analytical tools in medicine, food, bioprocessing, industry, and environmental monitoring, cell-based biosensors (CBB) have gained substantial attention as they are compact and provide a higher platform for biological activity. CBBs use bioactive compounds viz., enzymes, microorganisms, algae, fungi, bacteria, plant cells, etc., as the analyzer with an appropriate transducer attached to it, wherein most reported sensors depend on microorganisms such as bacteria and yeast [103]. Luminescent bacteria equipped with reporter protein are used in rapid food safety detection. The reporter gene exhibiting luminescent or colorimetric properties uses them as the detecting signal [104].

**Figure 11:**
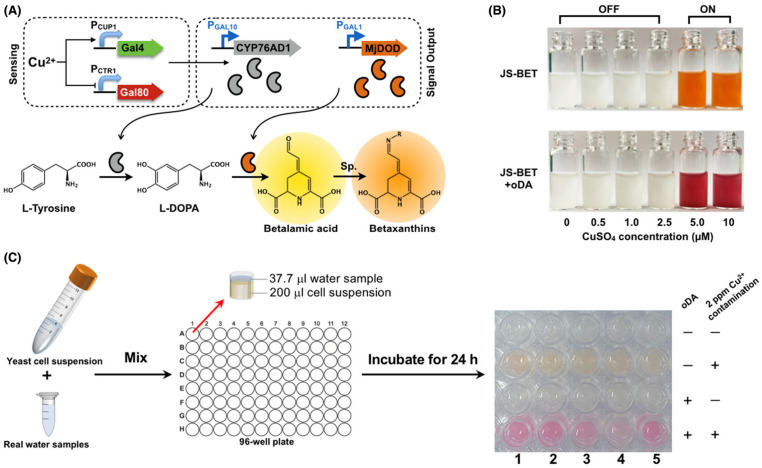
Betaxanthin-based biosensor for copper ion detection. (**A**) Schematic of copper ion detection, (**B**) detection using the biosensor phenotype, (**C**) various water sources with 2 ppm copper concentrations (reprinted with permission from Ref. no. [105], copyright 2022, John Wiley and Sons).

Fan et al. [105] engineered a *Saccharomyces cerevisiae* (yeast)-based biosensor for the on-site detection of copper ion (Cu(II)) (Figure 11). The sensor deliberately gives switch-like behaviour to respond with ‘yes/no’ for the presence of Cu(II) in the analyte. The sensor can respond with colorimetric output when integrated with the betaxanthin chromatic phenotype and can give styrene-based olfactory outputs when integrated with 2-phenylethanol. The detection of Cu(II) with betaxanthin-based colorimetric assay showed a limit of detection as low as 0.32 ppm. However, olfactory output required an intermediate complicated process of eliminating background odour resulting with 21.0 ± 1.48 mg/L of styrene which was beyond the human olfactory sensing limit.

**Figure 12:**
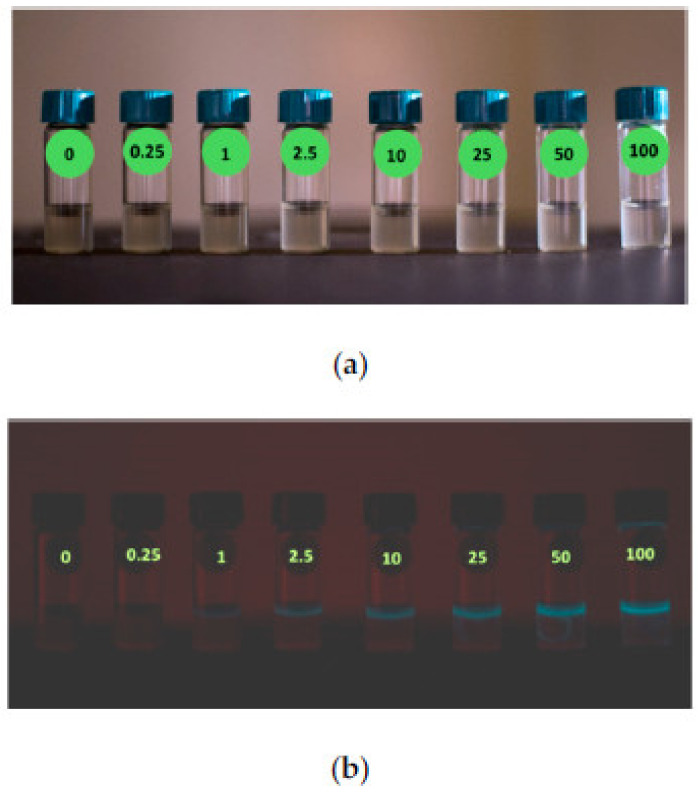
Photo images of the bioluminescence of the *E. coli* bacteria in mercury ion-treated water under different conditions: (**a**) light; and (**b**) no light (reprinted from Ref. no. [106], copyright 2019, MDPI).

Micro-level contamination of mercury (Hg^2+^) in water was assessed by employing a sensor based on bioluminescent *Escherichia coli*. *E. coli* strains were implanted within a photon sensitive small-dimension silicon photomultiplier (SiPM) optical detector [106]. Here, when the sensor encounters the presence of Hg^2+^; the luciferase protein in *E. coli* is induced to produce photon instantaneously giving out bioluminescence. An increasing trend in the bioluminescence was observed for increased concentrations of Hg^2+^ in the water sample as shown in Figure 12. Without additional reagent the developed biosensor was able to provide Hg^2+^ detection in water with an LOD down to 0.25 µg/L with a dynamic range of 0.25–25 µg/L.

### 5.6. COF- and MOF-Based Biosensors

Covalent organic frameworks (COFs) are crystalline materials developed from organic molecules with nano/microporous structures comprising covalent bonds. COFs are characterized by various advantages such as a completely conjugate structure, huge surface area, and one atomic thickness dimension. Hence, they find their potential applications in sensors, electrocatalysis, energy storage, and electrochemical devices [107,108]. Recent research has focused on the synthesis and design of COFs which belong to the category of porous crystalline organic nanomaterials owing to their inherent merits such as chemical and physical durability, excellent structural properties, strong covalent bonds, and high surface area due to porosity. COFs are typically applied in gas sensing, storage, and catalytic properties along with their use in sensing applications owing to their energy storage and optoelectronic properties. Utilization of COFs for the fabrication of biosensing applications was possible due to the short interlayer distance between the 2D structures which exists between the interactions between aromatic organic molecules within the layers [109,110,111].

Metal–organic frameworks (MOFs) are the materials with the most exciting architectures which initiates the fires of recent research through their excellent properties and applications [112]. MOFs are fabricated through the assemblage of multi-functional organic linkers and metal nodular clusters and possess a high level of porosity with a large surface area, and customizable and tunable characteristics. MOFs find most of their applications in biosensors, drug delivery, gas storage, biomedicines, biotechnology, biocatalysis, and bioseparation [113,114]. The application of MOFs has recently been extended to biosensing by a large amount for sensing of various analytes in various fields such as biomedicine, food, industry, and the environment. Electrochemical sensing has been the most common application of MOFs owing to their better insulation characteristics. When different molecules such as enzymes, antibodies, and aptamers are incorporated into MOFs, the scope of their applications in biosensing fields in the detection of proteins and DNA has been proven to expand further [115,116].

### 5.7. Other Nano-Materials in Biosensors

Transition metal oxides have been much explored recently for bio-sensing applications with their distinct electrochemical properties and variable oxidation states [117]. They are very well known for their cost-effectiveness and structure-dependent response. Oxygen atoms bound to the transition metal led to different polymorphs with variable stoichiometry [118]. The superior electrocatalytic activity, biocompatibility, and high surface area of Co_3_O_4_ has been taken up in designing a biosensor for glucose detection. The cubic Co_3_O_4_ crystals were fed as ink to screen-print the biosensing chip circuit. The chip exhibited a high sensitivity for glucose with a detection limit of 10 µM with a wide range from 10 to 600 µM [117]. TiO_2_, one of the most versatile metal oxides, has been effectively used in biosensor design with its photoelectrochemical properties. Tian et al. [119] reported the in situ integration of TiO_2_ nanotube arrays with CdS QDs for the detection of asulam (pesticide) in real environmental water samples. The fabricated sensor demonstrated a substantial linear range of 0.02–2.0 ng mL^−1^ with an LOD of 4.1 pg mL^−1^. ZnO nanorods grown on a SiO_2_/Si substrate provided good sensitivity for phosphate. The developed FET biosensor accomplished a sensitivity of 80.57 µAmM^−1^ cm^−2^ in a linear range of 1–7000 µM with an LOD of ~0.5µM [120]. A polymer-based composite between polyaniline nanosheets (PANINS) and NiO nanoparticles was prepared to bring out a non-glucose-detecting enzymatic biosensor [121]. The invented biosensor possessing a screen-printed NiO@PANINS electrode established a high sensitivity of 5625 μAmM^−1^ cm^−2^ with an LOD of 0.06 µM. The different nanomaterials used for the biosensor are presented in Table 1.

## 6. Applications of Nanotechnology-Based Biosensors

The development of biosensors has reached a tremendous altitude in various fields such as healthcare, bio-medicine, and food processing. The progression in nanotechnology with the development of various nanomaterials and nanotechnology goes hand in hand with the advancement of novel biosensors [122]. The ability of nanotechnology to control and regulate materials at their atomic and molecular level has opened avenues for a better understanding of their fundamental properties. The dimensionality of any material plays a vital role in determining its physicochemical and biological properties which assist in adopting them even in multidisciplinary applications [123]. The evolution of nano-biosensors set forth their application in biomedical diagnosis for monitoring and detection of the ultra-low concentration of analyte and the physicochemical phenomenon even in unreachable parts [124]. Biosensors are of different types based on the nature of their vital components such as bioreceptors and transducers.

Bioreceptors are the primary component of a biosensor based on which they are classified as enzymatic biosensors [125], immunosensors [126], aptamer biosensors [127], and microbial biosensors [128]. Secondly, based on transducer type, they are categorized as electrochemical biosensors [129], thermal biosensors [130], electronic biosensors [131], optical biosensors [132], and gravimetric biosensors [133]. Furthermore, based on the detection technology they are classified into nano-biosensors, surface plasma resonance biosensors [134], biosensors-on-chip [135], and electrometers [69]. With the remarkable progress established in the field of nanotechnology, biosensors will reach their pinnacle in biocompatibility, selectivity, sensitivity, wearability, and LOD in the upcoming years [136]. A few of the applications of biosensors are elaborated on in the following sections.

### 6.1. Biomedical Applications

The assessment of the progression in the health of patients is very crucial and needs precise monitoring of the condition of the disease to minimize the rate of mortality. Biosensors play a significant role in the biomedical field through multiple applications such as wearable devices, implantable devices, and so on to define the treatment protocol for the speedy recovery of patients. The bioresorbable pressure sensor designed by Shin et al. investigates the healing process of chronic disease and the pressure sensor is shielded by a SiO_2_ protective layer [137]. On the other hand, healthcare electronics are ruling the world presently. Sheng et al. fabricated a wearable flexible capacitor biosensor with many biomedical aspects such as accident alert, pressure measurement, remote control, and motion feedback [138]. Biodegradable polymers have emerged as a new class of biomaterials compatible with sensing applications. A few experimenters have manufactured an implantable biosensor for detecting glucose that could sense glucose catalytic reactions in a range of 0–10 mM [139]. Biosensors have created an optimistic revolution in the biomedical field. The specific biomedical applications of biosensors are discussed in the upcoming sections.

### 6.2. Cancer and Bone Disease

**Figure 13:**
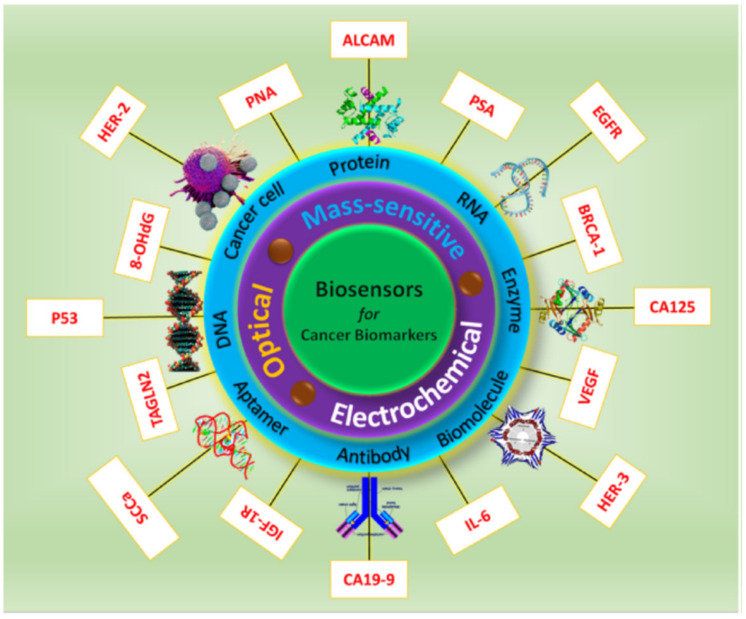
Schematic of biosensors’ working principle for cancer biomarkers’ detection (reprinted from Ref. no. [140], copyright 2021, Elsevier).

Cancer biomarkers (CBs) such as DNA, RNA, proteins, enzymes, and hormones that are released by genetic alteration are used as the essential aspect for screening the status of cancerous cells in patients. Detection of these CBs in the human body helps in undertaking relevant therapy with point-of-care applications [140]. Conventional techniques in practice for the detection of CBs impose technical limitations and require replacement with novel technology which is economically feasible and technically sound [141]. Premature cancer detection could highly reduce the rate of mortality and hence, it requires a highly precise tool for biomarker detection. Therefore, biosensors with their overwhelming properties have been advocated for cancer detection primarily in the last decade. According to Hasan et al., cancer-detecting biosensors are categorized as per the type of transducer and biological response as optical, mass-sensitive, and electrochemical sensors. Figure 13 depicts the workings of biosensors for cancer biomarker detection.

The enhanced biosensing by graphene through its property of easy binding with carbon-based rings present in biomolecules has been integrated with TiO_2_ for early-stage cancer detection [142]. This multilayer plasmonic sensor exhibited sensitivity for cancer cells from the skin, cervix, blood, adrenal glands and breasts with maximum angular sensitivity of 282.86 deg/RIU. Kim et al. [143] utilized Raman scattering as a tool for detecting breast cancer from human tears. This was realized by fabricating substrates with a gold-decorated, hexagonal-close-packed polystyrene (Au/HCP-PS) nanosphere monolayer and detection with a portable Raman spectrometer. In the same grade, bone diseases such as osteoarthritis and rheumatoid arthritis emphasize the essentiality of early diagnosis and treatment to elucidate the level of joint damage and inhibit added degradation of articular cartilage [144]. Hu et al. [145] proposed an SPR-based biosensor that could sense the miR-15a biomarker for the identification of rheumatoid arthritis with an LOD of 0.56 fM in a linear range of fM-0.5 nM. Similarly, a poly-hydrogel-film-based electrochemical biosensor was reported for the detection of osteoarthritis with an LOD of 2 nM with a linear range of 2–2000 nM [146].

### 6.3. Tissue Engineering Applications

**Figure 14:**
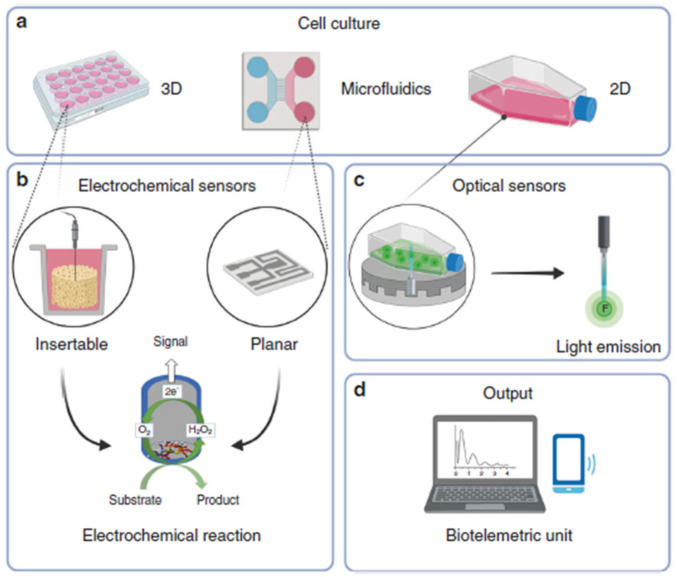
Applications of biosensors in the tissue engineering field using (**a**) electrochemical techniques, (**b**) the optical method, (**c**) real-time monitoring of quality, and (**d**) longitudinal studies (reprinted from Ref. no. [148], copyright 2021, Springer Nature).

The tissue engineering field is a versatile approach that could help in replacing damaged tissues involving the main biological components such as cells, scaffolds, and stimulatory molecules. This technology is very well received in terms of cost-cutting in comparison with organ/tissue transplantation [147]. The potential utilization of biosensors for tissue engineering and regenerative medicine is very minimum. In general, biosensors are used in the field of tissue engineering to ensure proper tissue growth by monitoring critical parameters such as oxygen/nutrient uptake and the release of metabolites [148]. An overall outlook on the usage of biosensors for the investigation of the quality of 3D constructs is explicitly shown in Figure 14. Because of the small dimension of biosensors, they can be relatively designed in desired shapes according to the cellular environment and can also be inserted inside tissues and organs. A platinum-wire-based enzymatic biosensor has been proven to be biocompatible and helpful in monitoring the quality of tissue implants when inserted into brain tissues [149]. Electrochemical sensors are useful for in vitro applications in monitoring the redox compounds that are released from the implants’ post-biological insertion. Indeed, in recent years, amperometric sensors have been applied in both in vitro and in vivo applications to analyze the quality of 3D constructs by sensing the release of different compounds such as dopamine [150], ascorbic acid [151], glucose [149], oxygen [152], and nitric oxide [153].

### 6.4. Microfluidic Systems

Microfluidic systems offer manipulation of sensing through a small volume of fluids that pass through lithographic microchannels. These lab-on-chip (LOC) devices can mimic the microenvironment around the conventional cell culture and help to isolate microparticles [154]. A biosensor integrated with a microfluidic system was designed for the diagnosis of breast cancer through the identification of an extracellular vesicle microRNA biomarker. The fabricated LOC captured breast cancer biomarker microRNA with a detection limit of 84 aM with range of 1 fM to 1 nM [155].

**Figure 15:**
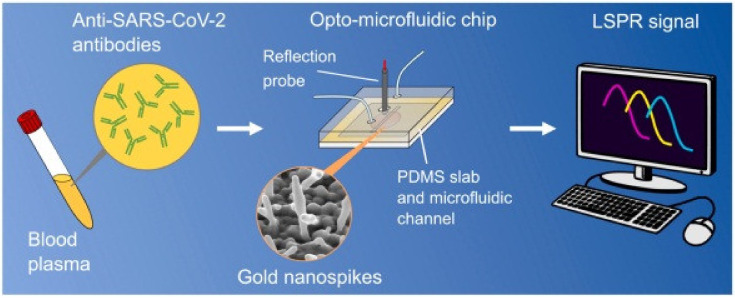
The opto-microfluidic chip with gold nanospikes (reprinted with permission from Ref. no. [156], copyright 2020, Elsevier).

A unique opto-microfluidic sensing platform was designed by a few researchers for detecting the COVID-19 virus at an early stage using human plasma, as shown in Figure 15. The sensing system was able to detect the COVID spike protein with a limit of detection of 0.5 pM and it consumes 30 min of time to complete the sample analysis [156]. An attempt to improve the sensing capability of an optical sensor was made by combining the merits of QDs with microfluidic technology. Two subtypes of the Influenza A virus (H5 and H9) could be identified by conjugating the antibodies with QDs. A bright fluorescence was produced by the QD-conjugated antibodies with an LOD of 0.26 and 0.017 HAU for H9 and H5, respectively [157].

### 6.5. Diagnostics

Biosensors hold huge potential for real-time microbial diagnostics. Biosensors when integrated with nanotechnology facilitate rapid, real-time, and accurate detection of molecular biomarkers in real samples. Researchers have focused on designing body-worn monitoring devices to acquire real-time diagnostic information using different label-free and lab-on-a-chip bioelectronic systems. A few experimenters constructed a stretchable electrochemical immune biosensor for the detection of tumor necrosis factor (TNF)-α which aids in wound heal monitoring. TNF immobilization was carried out using the differential pulse voltammetry method. The immunosensor shows a sensing performance with a clinical concentration range of (0.11 pM–0.09 µM) in human serum [158]. The contribution of electrochemical biosensors in detecting pathogenic bacteria has been elaborated by Karbelkar et al. [159]. The presence of *E. coli* in drinking water was detected by an electrochemical immune biosensor using a screen-printed electrode on a gold substrate with an LOD of 30 CFU mL^−1^ [160]. Rapid sensing of many pandemic and epidemic viruses using biosensing technology has been trending recently. Along the same line, a few researchers have developed a glycol nanoparticle-based immunosensor for the detection of the human influenza virus through SPR [161]. The study on the point-of-care detection of the Ebola virus in human survivors was undertaken by Brangel et al. [162]. The developed smartphone-based assay for the detection of IgG antibodies on infected patients in Uganda demonstrated 100% sensitivity and 98% specificity compared to the standard whole antigen.

### 6.6. Porphyrin and Phthalocyanine

Porphyrins (Pp) and pthalocyanines (Pc) are a special class of macrocyclic organic molecules possessing unique photophysical properties and hence find application as fluorescence probes in biomedical sensors, drug delivery, and fluorescence imaging. Their distinct optical properties originate from the arrangement of 18π electrons packed in a highly conjugated system. They have strong absorption towards the light where Pc absorbs mostly in the far infrared region [163]. Relaxation between different vibrational levels induces π–π* transition between the electrons in Pp giving rise to two types of fluorescence. The first type of fluorescence (S1-fluorescence) is observed in the range of 550–800 nm and is comparatively stronger than the second type. The other type (S2-fluorescence) of luminescence is weaker and observed between 400–550 nm [164]. The fluorescence property has been applied as a promising tool in various biomedical diagnoses. In this regard, a few experimenters fabricated a carboxyl Pp-based fluorescent biosensor to detect aflatoxin B1 present in medicines. Under optimization, the carboxyl Pp connected to the antigen could detect a least a value of 8.38 fg mL^−1^ in a linear range of 100 fg mL^−1^ to 100 ng mL^−1^ [165]. Furthermore, Attia’s group used the luminescence property of Pc for the determination of ovarian cancer antigen CA 125 in human serum samples. The biosensor designed with Ni-phthalocyanine doped in a polystyrene matrix was able to identify the cancer antigen as it quenched the intense fluorescence at 790 nm when excited with 685 nm. The investigation resulted in a detection limit of 1.0 × 10^−4^ U mL^−1^ in a linear range of application of about 1.0 × 10^−2^ −127 U mL^−1^ [166]. A porphyrin–Co_9_S_8_ nanocomposite synthesized via the hydrothermal route was applied for the detection of H_2_O_2_ through colorimetric sensing resulting in an LOD of 6.803 μM in a linear range of 7–100 μM [167].

### 6.7. Detection of Glucose

**Figure 16:**
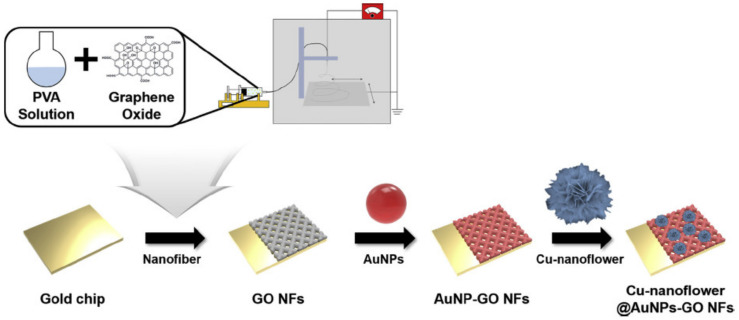
Schematic of Cu–nanoflower@AuNPs–GO nanofiber-based electrochemical nano-biosensor fabrication for glucose detection (reprinted with permission from Ref. no. [170], copyright 2020, Elsevier).

Biosensors have become a significant tool in the real-time clinical analysis of tahe wide variety of molecules present in bio-fluids, most significantly glucose. Glucose level sensing is essential for monitoring several complications such as diabetes, heart disease, high blood pressure, and kidney failure. Conventional sensing methods include the usage of enzymes, such as glucose oxidase, which has several limitations such as being highly expensive, having poor stability, and low reproducibility [167,168]. Hence, significant exertions have been made to develop a non-enzymatic electrochemical-based sensors. Ahmad et al. [169] proposed a novel CuO-nano-leaves-based electrochemical biosensor for glucose sensing. CuO nano-leaves were synthesized via a facile hydrothermal route and modified with a glassy carbon electrode. This non-enzymatic biosensor electrode displayed good stability and reproducibility with a high sensitivity of 1467.32 μA/(mM cm^2^) with a detection limit of 12 nM in a linear range of 0.005–5.89 mM. A gold-decorated hierarchical Cu nanoflower coated on GO nanofibers used as an electrode in the electrochemical biosensor (as shown in Figure 16) showcased excellent electrocatalytic properties in converting glucose to gluconic acid. During the experiment, the glucose concentration and current increased linearly along with each one facilitating the monitoring of glucose levels in the biofluids resulting in an LOD of 0.018 µM in a wider linear range of 0.001–0.1 mM [170].

A colorimetry-based biosensor with nanolayered manganese–calcium oxide mimicking the enzymatic behavior of glucose oxidase was engineered by Rashtbari et al. [171]. Colorimetric strategy was used for the on-time simultaneous detection of glucose and H_2_O_2_ from a human serum sample. The oxidation reaction taking place to convert glucose into gluconic acid in the presence of O_2_ and MnCaO_2_ changes the color of the reaction solution to red-orange facilitating the naked-eye detection of glucose by spectrophotometry. The experiment resulted in the identification of glucose with an LOD of 6.12 × 10^−6^ M in a linear range of (0–82.3) × 10^−6^ M.

### 6.8. Detection of DNA and Protein

Biosensors are used as an analytical tool for the detection of biomarkers such as DNA and proteins. In a biosensor, the resultant signal from the reaction of biological components is converted to measurable signals by a transducer. These biological components are usually protein biomarkers that underlay the existing condition of a disease. A few experimenters have manufactured a label-free and sensitive optical biosensor with Si nanowire to detect C-reactive protein (CRP) present in the human serum. Here, the surface of the nanowire was modified with a CRP antibody and the LOD for the immunosensor was achieved down to 1.6 fM [172]. Pork content present in the edible items was detected by a gold–DNA-conjugated electrochemical sensor [173]. The sensor detected the existence of pork in food by recognizing the presence of Sus scrofa mtDNA (pig DNA) in various raw and processed meat samples with an LOD of 0.59 μg/mL. Some experimenters stated the design of a biosensor for the first time to detect the TAR DNA binding protein, a biomarker for neurodegenerative disorders. The bioconjugation method for the fabrication of biosensors helped in cutting down the cost and gave rapid detection of DNA in a detection time of 1 h [174]. The nanomaterials used for various biosensing applications are compiled and listed in Table 2.

## 7. Challenges and Prospects

**Figure 17:**
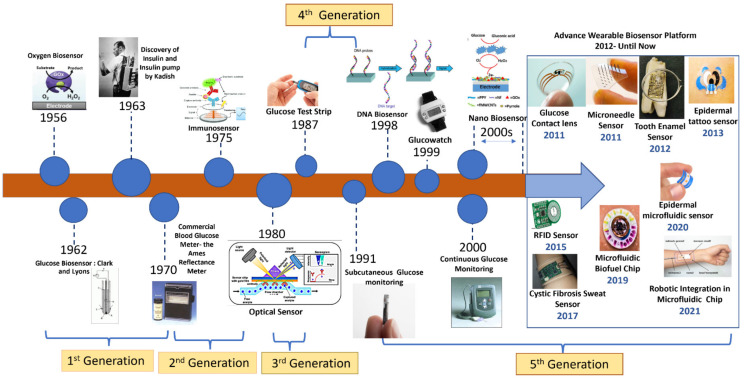
Biosensor development timeline (reprinted from [180], copyright 2022, MDPI).

In terms of the difficulties associated with translating in vitro systems to in vivo systems, nanobiosensors are now a fundamental aspect of research. The interconnectedness of the system’s parameters has a profound effect on the expression of different analytes, making systematic monitoring in real time an absolute need. Many industries are taking advantage of recent breakthroughs in the field of nanobiosensors owing to their excellent magnetic, electrochemical sensors, acoustic, piezoelectric, and optical properties, but tissue engineering has yet to overcome the significant barrier of incorporating these sensors [41,42,53]. No one can deny nanotechnology’s impact in propelling biosensors to new heights. It has been demonstrated that using nanomaterials/nanostructures for biosensing applications can increase important sensor properties, including LOD, precision, and reliability. Such cutting-edge biosensors have been shown to rapidly detect the target analyte, exhibit single-molecule detection, and considerably boost transduction outputs. The obstacles to biosensors’ widespread use have been eased thanks to these characteristics. However, there are also drawbacks to this technology, including the unavoidable emission of nanoparticles further into the atmosphere [84,94]. In addition, quantum effects produce exceptionally high sensitivity, random noise, and background signals. The response of such sensor exposure can lead to certain analytes that have been observed to be cross-sensitive, nonlinear, and unpredictable. Materials such as graphene are promising for biosensing applications; however, they have not been effectively mass-produced. The world will not be able to fully use nanotechnology’s incredible potential in biosensors unless these issues are resolved [104,121]. Figure 17 shows the timeline of developments in the field of biosensors. Development began with the advent of oxygen-based biosensors and recently QDs have been used for the development of biosensors. This development timeline is expected to enter into the field of tissue engineering and other allied biomedical fields where the advent of nanobiosensors might make a larger difference in comparison to the current system of biosensors [180,181].

The use of ML represents one of the innovative technologies that is currently being applied to the problem of such shortcomings. When considering biosensor implementations, ML could be considered as an algorithmic strategy for examining sensor data and determining usable information via statistical methods [145]. Traditional applications of ML have been in the areas of classification and regression. Naturally, then, such resources are of great use in the discipline of chemometrics. Support-vector machines, random forests, artificial neural networks, convolutional neural networks, Naive Bayes, and k-nearest neighbors are a few of the most popular machine learning algorithms a few of the most popular machine learning algorithms in use for this purpose. Diverse researchers have elaborated [146] on the deeper perspectives of using ML algorithms for biosensing applications and their data processing. Their superior pattern recognition capabilities and machine learning algorithms, with their superior pattern recognition capabilities, can help nano-biosensors draw insights from raw data. Examples of applications of such algorithms include the classification of raw sensor data and the mitigation of cross-sensitivity and misclassification. Reduced detection limits are possible thanks to the application of ML algorithms for filtering out irrelevant data from the sensor output.

Biomarker methods will investigate the escalating possibilities for developing targeted therapeutics, diagnostic tools, and medical equipment. There is room for innovative change in the way human samples are gathered. Surgically implanted biosensors may play a significant role in hastening the development of individualized therapeutics. They will let scientists keep a close eye on the outcomes of potential new treatments in the body, allowing them to more properly gauge whether a drug can proceed to clinical testing. In addition, biosensor chip technology can be implanted to detect complex DNA alterations in the blood before the onset of illness symptoms. Biosensor innovation can be utilized in reversible and low-cost care point devices. It also has the capability to monitor implanted devices in real-time. Smart bracelets included in wearable devices can non-invasively monitor collected samples such as saliva and expelled condensing breath and invasively collected samples such as blood and interstitial fluid [151,160].

There are several important technical issues that must be resolved, such as extending the lifespan of the sensors. Biosensors provide a mechanistic understanding of biological structures down to the molecular level. Numerous fields, including the analysis of biological processes, agriculture, medicine, and environmental technology, have found numerous applications for these kinds of analysis instruments. As a result of this understanding, numerous approaches have been developed for identifying biomolecules; they serve crucial roles in many areas of biotechnology, including drug discovery and targeting, pathogen detection, gene therapy, and many others. The need for biosensors is increasing because of their widespread application in healthcare and medicine. In addition, advances in biosensors’ use across various fields—including human health management, patient health surveillance, diagnosis, and illness detection—have paved the path to rapid expansion in this field [53,58,124].

Another possibility is wearable biosensors in conjunction with ML for health monitoring. On account of their immense potential for the non-invasive evaluation of human physiology in a broad range of biological fluids, wearable biosensors have garnered considerable attention. Wearable biosensors’ goal is to continually monitor biomarkers by integrating a succession of sensing devices on flexible patches. Multiplexed sensory data can be analyzed using ML to determine a patient’s health status from time series data. For these uses, ML must be transparent. Healthcare providers and policymakers must be able to grasp the algorithm’s verdict. Meanwhile, a deep training process must have transparent human knowledge with reasoning principles transparently integrated into it to control and enforce its learning and decision-making. Furthermore, the size of the samples needed for ML algorithm training can also be drastically reduced with the help of manual overriding. This highlights the critical necessity to integrate explainable ML in the field of wearable electronics in healthcare applications and related medical actions [37,168].

## 8. Summary and Conclusions

Nanotechnology-enabled biosensor technology has been comprehensively discussed in the above sections. ML techniques can enhance the quantitative forecasting of trace analytes, and qualitative discrimination of complicated overlapping signals can also be accomplished. Deep learning techniques such as convolutional neural networks (CNNs) and recurrent neural networks (RNNs) in sensory data analysis are specifically witnessing a rise. Regression analysis with traditional data requires a different formula to determine the sample’s dependent variables. Typically, there are fewer than two features used as inputs. In contrast, state-of-the-art ML models can handle a database with hundreds of input data. A large enough data set is required for deep learning techniques to work. Researchers can get beyond the data bottleneck between ML and biosensors by designing and using multiplex or high-throughput biomaterial sensors such as microarrays and channel fluidic chips.

Biosensors have many uses in the medical profession and are of great benefit to both patients and clinicians for a variety of reasons, including disease prevention and management, clinical diagnosis and treatment, access to health records, and evaluation of treatment outcomes. Nanomaterials have recently demonstrated extensive use in the creation of biosensors. The primary goal of clinical medicine is to classify patients into different categories using simple biosensors. There is widespread consensus that biosensors make possible tailored medicine possible, which represents a radical departure from conventional medical practice. This method has profoundly impacted the healthcare industry, which has led to numerous therapeutic and diagnostic options. Many studies’ findings point to LC interface concepts that could be applied to constructing stimuli-responsive materials for highly precise biosensors. The target molecules, immobilization methods, and enzymes, chosen will all play a role in how fruitful this field proves to be.

Several smart nanostructure-based techniques could be implemented to boost the LC biosensor’s sensing performance—particularly its low detection limit and high sensitivity. Moreover, such sensing systems are inexpensive since they can be easily fabricated without the need for costly laboratory equipment, they require little operational power, and their sensing results are consistent and repeatable even when performed on different batches. This article has summarized the methods and mechanisms used to create portable LC-based biosensors for label-free detection of a specific analyte at a low concentration. The authors are committed to complete further research into using such sensors for illness diagnosis, food safety, and the control of epidemics. We think that nano-enabled LCs-biosensors have the potential to be used for recognizing any biological substances because of their desirable sensing performance.

## Figures and Tables

**Table 1:** Nanomaterials used in biosensors for the detection of various analytes.

Material	Target Analyte	Methodology	LOD	Detection Range	Ref.
Graphene	ZIKA NS1	FEB technology	0.45 nM	-	[74]
Graphene	Living cells	Refractive index changes	1.2 × 10^8^ mV/RIU	-	[81]
Graphene	SARS-COV 2	FET biosensing	1 fg/mL	-	[82]
Graphene	COVID 19	Receptor binding domains	0.2 pM	-	[83]
rGO	E7 protein of HPV	Electrolyte gated FET	100 pg/mL	-	[84]
GNP	Protein biomarker	Micro-cantilever technology	21 pg/mL	-	[91]
GNP	Gene allergic to peanut, sesame, and soybean	Colorimetric	0.5 nm	-	[89]
GNP	SARS-COV2	Dual sensing- Colorimetric Electrochemical	48 ng/mL 1 pg/mL	-	[90]
Graphene-GNP	Hepatitis-B	π-π stacking of ssDNA	50 pg/mL	-	[92]
CNT-PAN	Microbacterium Tuberculosis	Electrochemical biosensing	0.33 fm	-	[95]
CNT	Lactase	Fabric-based wearable gloves	1.4 nm	-	[96]
CNT/PLA	Thermal injuries	Microneedle array matrix	180 µm	-	[97]
CQD	Acrylamide	Fluorescence	2.4 × 10^−8^ M	-	[99]
ZnO QDs	Cysteine	Fluorescence	0.642 µm	0.1 µm–600 µm	[100]
GQD-GNP	Lung cancer	Fluorescence	0.09 pg mL^−1^	0.11 pg mL^−1^ to 1005 ng mL^−1^	[101]
PB-CdSe QD-rGO QD	Glucose and H_2_O_2_	Wearable sensor	37.26 µA/mM.cm^2^ (Glucose) 53.8 µA/mM.cm^2^ (H_2_O_2_)	-	[102]
*Saccharomyces cerevisiae*	Cu(II)	Colorimetric and Olfactory output	0.322 ppm and 21.0 ± 1.48 µg/mL	-	[105]
*Escherichia coli*	Hg^2+^	SiPM optical detector	0.25 µg/L	0.25 to 25 µg/L	[106]
Co_3_O_4_	Glucose	Electrocatalytic	10 µM	10–600 µM	[117]
TiO_2_/CdS QDs	Asulam	Electrochemical	4.1 pg/mL	0.02–2.0 ng mL^−1^	[119]
ZnOnanorods	Phosphate	FET biosensing	~0.51 µM	0.11 µM–7.2 mM	[120]
NiO@PANINS	Glucose	Amperometry	0.06 µM	-	[121]

**Table 2:** List of nanomaterials used for various biosensing applications.

Nanomaterial	Applications	Target Analyte	Methodology	LOD	Detection Range	Ref.
Si nanowire	Cancer detection	8-OHdG	Electrochemical	1 ng/mL	-	[175]
GO	Cancer detection	PEAK1	Electrochemical	10 pg/mL	-	[176]
TiO_2_/Au/graphene	Cancer detection	MCF7	Surface Plasmon Resonance	292.86 deg/RIU	-	[142]
Au/HCP PS	Cancer detection	2-NAT	Surface enhanced Raman scattering	100 fM	10^−8^–10^−3^ M	[143]
SPRi-Gold chip	Rheumatoid arthritis	miR-15a	Surface Plasmon Resonance	0.56 fM	5 fM–0.5 nM	[145]
Poly hydrogel film	Osteoarthritis	Proteases	Electrochemical	2 nM	2–2000 nM	[146]
Silver nanoparticle	Kidney tissue	MCF-7	Aptamer conjugation	-	-	[177]
Graphene/gold	Liver metastasis	PHD-1	Electrochemical	4 μM	-	[178]
nMgO	Periodontal tissue regeneration	-	GTR	-	-	[179]
Zeolite	Neuro transmitter	Dopamine	Amperometry	-	-	[150]
Nanoporous gold	Fungus detection	Ascorbic acid	Electrochemical	2 μmol L^−1^	-	[151]
Carbon	Brain tissue	Oxygen	Biotelemetry	≤5 M	-	[152]
-	Cancer detection	microRNA	microfluidic	84 aM	1 fM–1 nM	[153]
Gold nanospike	COVID-19	Cov 2 spike	microfluidic	0.5 pM	-	[154]
-	Influenza A	H 5 H 9	microfluidic	0.016 HAU (H5) 0.25 HAU (H9)	-	[155]
Ni-Phthalocyanine	Cancer detection	CA125	Optical sensing	1.0 × 10^−4^ U mL^−1^	-	[163]
Co_9_S_8_-Porphyrin	H_2_O_2_ detection	-	Colorimetry	6.803 μM	7–100 μM	[166]
NiO	Glucose detection	Saliva	Electrochemical	~84 nM	~5 µM to 0.825 mM	[168]
CuO nanoleaves	Glucose detection	-	Electrochemical	12 nM	0.005–5.89 mM	[169]
Au/Cu/GO	Glucose detection	-	Electrocatalysis	0.018 μM	0.001–0.1 mM	[170]
MnCaO_2_	Glucose detection	Human serum	Colorimetry	6.12 × 10^−6^ M	(0–82.3) × 10^−6^ M	[171]

## Data Availability

Not applicable.
